# Psychosocial moderators of the effect of lifestyle interventions in primary prevention of cardiovascular disease: a scoping review

**DOI:** 10.1186/s12889-025-24076-2

**Published:** 2025-08-30

**Authors:** John Andersson, Melony Fortuin De Smidt, Anna E Sundström, Steven Nordin, Patrik Wennberg, Ulf Näslund

**Affiliations:** 1 Department of Psychology, Umeå University, Umeå, Sweden; 2 Department of Public Health and Clinical Medicine, Umeå University, Umeå, Sweden

**Keywords:** Behavior change, Psychosocial, Moderator, Lifestyle modification, Public health, Health behavior

## Abstract

**Background:**

Lifestyle modification plays a key role in prevention of cardiovascular disease (CVD), but often fails due to non-adherence to lifestyle recommendations. Previous research has highlighted the importance of psychosocial factors in non-adherence, though focused on *secondary* rather than *primary* prevention. The aim of this scoping review was to provide an overview of the moderating role of psychosocial factors on the effect of lifestyle interventions in primary CVD prevention.

**Methods:**

A literature search of scientific databases was performed to identify studies published in peer-reviewed journals, that evaluated lifestyle interventions in primary prevention in adult populations (18 years and older), with a composite CVD risk score or a CVD risk factor (diet, physical activity, smoking or alcohol) as outcome, and assessed the moderating effect of a psychosocial factor.

**Results:**

Thirty-five studies published between 2000 and 2025 were included in this review. Most were randomized controlled trials (RCT), included middle-aged participants, and investigated samples in which women were in majority. The outcomes differed, with fourteen studies reporting on physical activity, eleven on diet, six on body weight, two on smoking and one on alcohol. One study used a CVD risk score as the outcome. The studies included a broad array of psychological factors that were grouped into five categories: self-efficacy or motivation (*n* = 11), social support or relationship quality (*n* = 8), mental health (*n* = 6), personality or emotions (*n* = 6), and cognitive factors (*n* = 4). Sixteen (44%) of the studies did not use validated instruments in the assessment of the psychosocial factor.

**Conclusions:**

This review highlights the potential role of psychosocial factors on the effectiveness of lifestyle interventions. However, our ability to draw detailed conclusions or identify any general trends were limited by the heterogeneity amongst the studies in terms of study design, assessment of outcomes and moderators, and populations. Still, we identified a lack of RCTs (1) with long follow-up time, (2) with sufficient sample size, (3) using validated instruments to assess the psychosocial moderator, and (4) using interaction analyses to assess moderating effect.

**Trial registration:**

This scoping review was registered at Open Science Framework (osf.io) under 10.17605/OSF.IO/VEADK on December 19, 2023.

**Supplementary Information:**

The online version contains supplementary material available at 10.1186/s12889-025-24076-2.

## Background

Cardiovascular disease (CVD) is a common cause of death among people who have not yet reached the age of average life expectancy [1]. Over the past decades, CVDs such as myocardial infarctions and stroke have dominated global disease burden in terms of prevalence, mortality, and disability-adjusted life years [2–4]. Fortunately, atherosclerosis, the main underlying process of CVD, can be slowed down and even reversed. As many as 80–85% of all CVDs are related to modifiable lifestyle factors [5, 6] such as lack of physical activity [7], unhealthy diet [8], and smoking [9]. As a consequence, lifestyle modification has become the main approach in CVD prevention, accompanied by pharmacological treatment of hyperlipidemia and hypertension [10].

Still, prevention often fails due to non-adherence to healthy lifestyle recommendations [11]. The World Health Organization defines adherence as the extent to which a person’s behavior, including taking medication, following a diet, and/or executing lifestyle changes, corresponds with agreed recommendations from a healthcare provider [12]. It has been suggested that adherence to a healthy lifestyle has the potential to reduce the incidence of coronary heart disease by 80%, and of ischemic stroke by 50% [13].

Adherence to CVD preventive guidelines could be strengthened by personalized medicine, i.e., the tailoring of medical treatment to a person’s individual characteristics, including psychosocial factors. In a position paper, the German Cardiac Society argues that psychosocial risk factors are not only highly prevalent among people with CVD, but are also associated with behavioral and biological risk factors for CVD, morbidity and mortality in CVD and low quality of life [14]. Psychosocial factors like depression and anxiety may hinder behavior change and adherence to medication [14]. Since these conditions may involve lower energy levels, impaired executive functioning and increased avoidance behavior [15]. Conversely, other psychosocial factors, for example self-efficacy, can facilitate behavior change through a variety of psychological, behavioral, and social mechanisms [16].

An extensive consensus statement from the European Association of Preventive Cardiology concluded that optimal patient adherence to treatment is a key factor in successful secondary prevention of CVD and provided support for the relevance of psychosocial factors (e.g. self-efficacy, risk perception, cognition, mental health and social support) for adherence to interventions in secondary CVD prevention [17].

Adherence in terms of individuals’ attendance to, and completion of, lifestyle intervention programs, is affected by practical, social and psychological factors [18–20] Although studies focusing on attendance provide valuable information, it is important to evaluate psychosocial factors in relation to actual behavioral change, and the maintenance of these behaviors over time. In three systematic reviews, Murray and colleagues [20–22] summarized results, mainly from qualitative studies, on the influence of *perceived* barriers and facilitators on uptake and completion of lifestyle modification programs for secondary CVD prevention [20, 22] as well as maintenance of healthy behaviors in individuals at high risk of cardiovascular disease [21]. Their results show that psychosocial factors such as social support, beliefs regarding illness and lifestyle change, and mental ill-health (e.g. depression and anxiety) were consistently associated with uptake of lifestyle change [20] and are key factors for maintaining lifestyle change over time [21].

Many of the aforementioned studies have focused on the role of psychosocial factors in *secondary* prevention, i.e., interventions for people recovering from or previously diagnosed with CVD. Less is known about their role within *primary* prevention. Since atherosclerosis can begin early in life [23], promotion of a healthy lifestyle targeting asymptomatic individuals is essential to improve health and reduce the burden of cardiovascular disease. However, implementing such interventions in people without CVD may be more challenging and less effective than in secondary prevention [24]. Understanding the factors that influence the success of lifestyle interventions in primary CVD prevention is therefore crucial for tailoring programs to individuals or specific subgroups. Moreover, factors moderating behavior change in asymptomatic individuals or those with low perceived CVD risk - such as younger adults - may differ from those affecting individuals with established CVD. From a public health perspective, primary prevention is critical, as most people who develop CVD come from low- or moderate-risk groups, while only a minority belong to the high-risk population due to its smaller size [25]. This apparent contradiction, often referred to as “the prevention paradox,” highlights the importance of population-wide lifestyle interventions.

To our knowledge, no previous review of systematic character has examined the moderating effects of psychosocial factors on lifestyle interventions in primary CVD prevention. Addressing this gap, the present review focuses on identifying psychosocial moderators in interventions targeting individuals without CVD.

### Objectives

The aim of this scoping review was to provide an overview of the moderating role of psychosocial factors on the effect of lifestyle interventions in primary CVD prevention. More specifically, the was to systematically chart all quantitative studies, published in peer-reviewed journals, that in primary prevention have investigated psychosocial factors as moderators of the effectiveness of interventions intended to lower CVD risk by changing relevant outcomes such as dietary habits, physical activity, smoking, or alcohol consumption. It also included interventions aimed at changing scores on algorithms such as the Framingham Risk Score (FRS), Systematic Coronary Risk Estimation or Systematic Coronary Risk Estimation (SCORE) and (SCORE2). The purpose was to visualize the range of knowledge that is available as well as to identify important knowledge gaps regarding all adult ages. A scoping review approach was chosen because it allows for exploration and synthesizing complex and diverse evidence in the literature [26], and this type of review was considered appropriate to summarize research on a broad scope of psychosocial moderators as well as a variety of outcome measures.

## Methods

### Protocol and registration

A full protocol of this scoping review was registered at Open Science Framework (osf.io) under 10.17605/OSF.IO/VEADK on December 19, 2023. The Preferred Reporting Items for Systematic Reviews and Meta-Analysis extension for Scoping Reviews (PRISMA-ScR) checklist was used for reporting [27].

### Selection criteria

Original empirical studies of quantitative character that were published in English in peer-review journals were eligible for inclusion. The Population (or participants)/Concept/Context framework was used to construct clear and meaningful objectives and eligibility criteria for a scoping review [28]:

- Population: Adults from the age of 18 years without CVD.
- Concept: Psychosocial factors assessed as moderators of the effect of lifestyle interventions.
- Context: Lifestyle modification interventions in primary prevention/primary care.

### Information sources

A researcher (JA) conducted searches in the databases PubMed, Web of Science, APA PsycInfo, and Scopus. The first search was conducted on November 29, 2023. The database search was rerun before final compilation of the results on May 7, 2025, to include recently published articles.

### Search strategy

The psychosocial moderators of interest for this scoping review were *Personality-related factors*: Personality, optimism, pessimism, vitality, esteem, confidence and attitude; *Emotional factors*: Emotions, affects, mood, feelings, attachment, anger, hostility, gratitude, happiness, resilience, hope, helplessness and hopelessness; *Factors related to physical and mental health*: Mental health, attitudes, worry, anxiety, depression, body-dissatisfaction, quality of life, well-being, health beliefs and life-satisfaction; *Factors related to behavior change*: Coping, motivation, self-efficacy, self-regulation, readiness for change and response-efficacy; *Factors related to stress*: Stress, distress, post-traumatic stress disorder, trauma, exhaustion, burnout and fatigue; *Cognitive factors*: Cognition, executive functions, memory, intelligence, health literacy and risk perception; and *Factors related to social interactions*: Social support, social networks, loneliness and norms. The choice of search terms related to possible moderators was guided by a clinical consensus statement [17] which discusses strategies for optimizing adherence in secondary CVD prevention. According to the consensus statement, patients’ ability to adhere to behavioral changes and medical treatment depends on complex cognitive-emotional capacities and social interactions. Therefore, cognitive, emotional, mental, motivational, and personality factors were important to consider. These categories served as starting points for generating search terms for psychosocial moderators. From there, subsequent search terms emerged through discussions among the authors, and further expanded upon by finding synonyms, related terms and alternate spellings.

The search strategy was developed in collaboration with two information specialists at the Umeå University Library. The search period was from 1983 to the present, as pilot searches in these databases did not identify publications prior to 1983. The search strings used in searches of titles, abstracts, and (where applicable) keywords are given in Appendix A. Search syntaxes and descriptions of applied search filters for the different databases are given in Appendix B. In short, the search string was designed to identify articles that included [1] an outcome of interest, e.g. *“physical activity”* or *“cardiovascular risk”* [2], a term related to behavior change, e.g. *“behavior change”* or *“lifestyle modification”* [3], a psychosocial factor, e.g. *“self-efficacy”* or *”anxiety”*, and [4] a suitable research design, e.g. *“randomized controlled trial”* (RCT) or *“longitudinal”.* Rayyan (https://rayyan.qcri.org/), a web-based tool for screening and selecting studies in systematic reviews [29], was used as a platform where the reviewers manually scanned all abstracts.

### Selection process

Inclusion criteria were (a) peer-reviewed, original quantitative empirical studies (b) published in English, because the vast majority of scientific articles are written in English and because English is the only language all three authors are fluent in, (c) studies reporting on adult populations from 18 years of age, (d) longitudinal studies reporting on a lifestyle- or behavior-change intervention in primary prevention, (e) studies with any of the following outcome variables: physical activity, smoking, body weight, alcohol consumption, dietary (healthy eating, caloric intake, etc.), Framingham Risk Score (FRS), Systematic Coronary Risk Estimation (SCORE) or Systematic Coronary Risk Estimation 2 (SCORE2), (f) studies assessing the relationship between one or more psychosocial factor(s) and the outcome. More specifically, we included studies that investigated whether a baseline psychosocial factor was a moderator of the intervention effect, evaluated by assessing the interaction between the psychosocial factor and study group (intervention vs. control) and/or subgroup analyses.

Exclusion criteria were (a) studies on secondary prevention (e.g. populations who have had or are recovering from CVD) (b) studies conducted on populations with health conditions not of clear relevance for CVD (e.g. interventions aiming to increase physical activity in people with chronic pain, and (c) studies evaluating psychosocial factors as predictors of lifestyle change, regardless of intervention-group.

In the process of screening reports for identification of eligibility, three reviewers (JA, MFDS and AES) initially conducted a calibration exercise based on titles and abstracts which involved reviewing twenty abstracts and checking for coherence among the reviewers. In this exercise the reviewers initially disagreed on four abstracts. After a discussion to reach coherence, this procedure was repeated with 20 other abstracts after which the reviewers agreed on all. Thereafter, all identified publications from the search were assessed for eligibility in an initial screening phase based on titles and abstracts. In the first screening phase, each report was screened by one of the reviewers (JA, MFDS or AES). In cases where the reviewer could not decide single-handedly, all three reviewers screened and discussed the report until a consensus decision could be made. If questions about a report remained, these were also discussed with SN. In the second screening phase, all three reviewers reviewed the abstracts of all reports that had passed the first screening phase. In addition, at least one reviewer read the methods and results sections of each full-text article.

### Data charting process

An a priori produced data-charting form was used. This included (a) author(s), publication year, and study location, (b) intervention, study design, and follow-up time (c) outcome and its assessment, (d) psychosocial factor and its assessment (e) study population and characteristics and, (f) type of analyses and main findings. As a scoping review, and apart from the brief critical appraisal, no formal risk of bias assessment was conducted for the included studies. The basis of the critical appraisal was limited to the information provided in the data-charting form and included an assessment of limitations in terms of design (e.g., lack of control group), sample size, recruitment and sample representativeness, as well as attrition and reliability and validity of measures used.

### Data synthesis

To facilitate the reporting of the results, the included studies were organized into thematic categories based on the psychosocial factor assessed as a moderator. This categorization closely resembled the initial categorization of moderators used for the search string presented in the introduction and comprised the following categories: Cognitive factors, Mental health, Motivation and Self-efficacy, Personality and Emotions, and Social Support and Relationship Quality. Due to the small number of studies on stress as a moderator, these studies were incorporated in the Mental health category. Thereafter, a scoping review was conducted based on the outcomes, age of participants and main findings. The design of each study was identified according to published design descriptions [30].

Interventions that were used in the studies were described based on the inclusion of the following components: (1) health education/information, (2) a specific diet or exercise programme, (3) behavioral counselling, (4) goal setting and planning, and (5) feedback and monitoring. An intervention was categorized into the last two categories only if it aligned with the Behavior Change Techniques (BCTs) noted in the BCT Taxonomy (BCTTv1) [31] Group 1 (“Goals and Planning”) and Group 2 (“Feedback and Monitoring”). It is important to note that an intervention can include multiple components, and that an in-depth categorization based on all the BCTTv1-groups was not feasible.

## Results

### Selection of studies

**Fig. 1:**
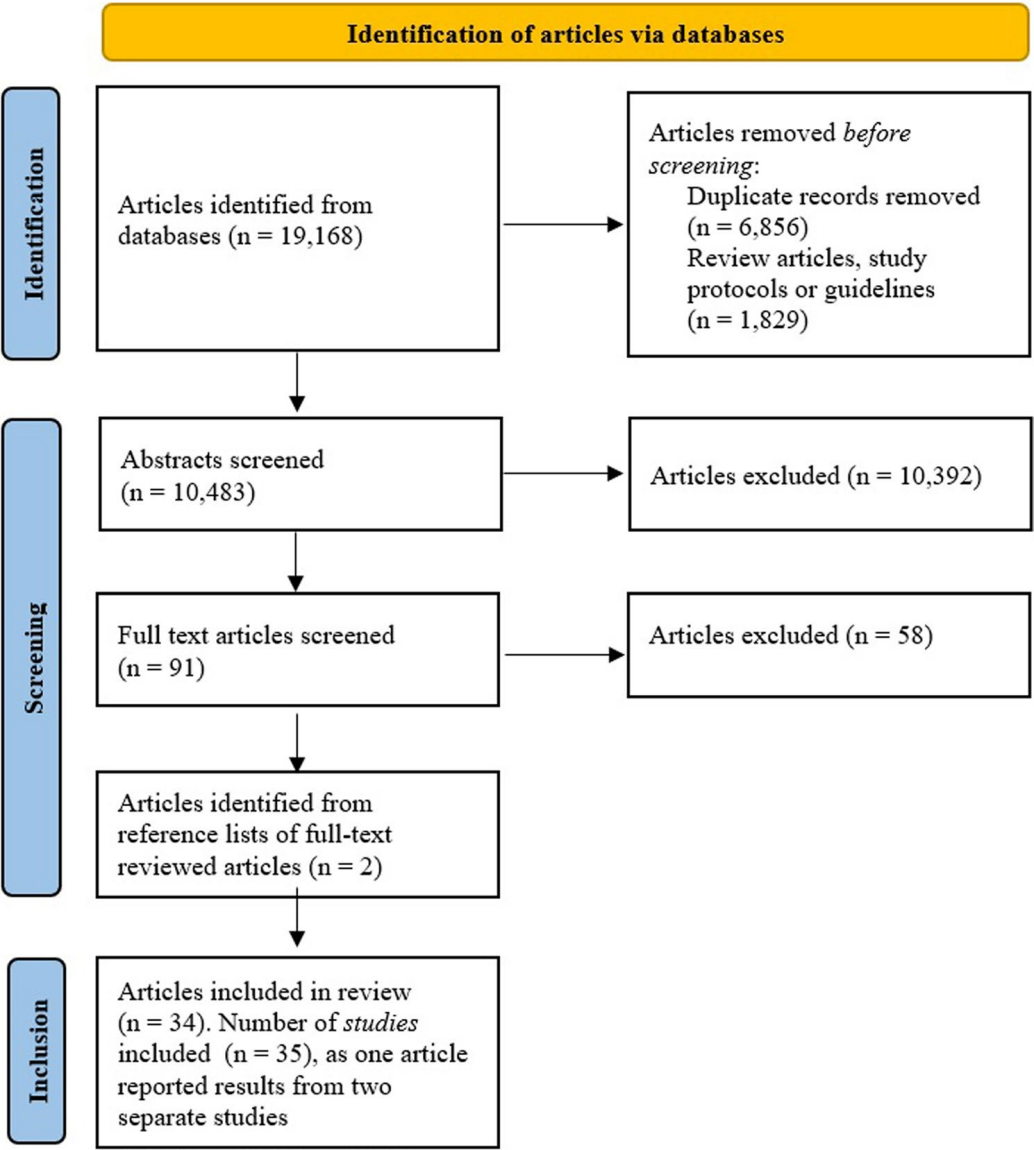
PRISMAflow diagram of the search results and the included articles through the screening process

The number of studies screened, assessed for eligibility, and included in the review, with reasons for exclusion at each stage, is presented in Fig. 1. Of 19,168 records identified from the databases, 10,438 were screened in the first phase, resulting in 91 studies being retrieved. After reviewing the full text versions of these 91 studies, 33 articles were included in the review. Finally, two additional articles were identified from reference lists of full text articles and subsequently included, bringing the total number of included articles to 35, all published between 2000 and 2025. One article included two studies and therefore, the number of studies included was 35. Overall characteristics of the studies included are presented in Table 1. Summaries of the individual studies, categorized by the psychosocial factor being investigated, are presented in Tables 2, 3, 4, 5, 6. Note that studies may have investigated multiple outcomes, multiple psychosocial factors, and multifaceted interventions.

**Table 1:** Characteristics of included studies

Characteristic	Studies *n* (%)
Outcome	
Physical Activity	17 (49)
Diet	12 (34)
Body Weight or BMI	5 (14)
Smoking	2 (6)
Alcohol Consumption	3 (9)
CVD Risk Score	1 (3)
Category of Psychosocial factor	
Self-Efficacy or Motivation	11 (31)
Social Support or Relationship Quality	8 (23)
Mental Health	7 (20)
Personality or Emotions	6 (17)
Cognitive factors	4 (11)
Type of intervention	
Health education	16 (46)
Exercise- or diet program	3 (9)
Behavioral counseling	16 (46)
Goal setting and planning	24 (69)
Feedback and monitoring	15 (43)
Study Design	
RCT	26 (74)
Cluster randomized trial	4 (11)
Quasi Experiment	3 (8)
Prospective study with control group	1 (3)
Prospective study without control group	1 (3)
Follow-Up Time	
< 1 month	4 (11)
> 1–6 months	14 (40)
> 6–12 months	7 (20)
> 12 months	10 (29)
Sample Size	
< 100	5 (14)
101–500	21 (60)
501-1.000	4 (11)
> 1.000	5 (14)
Mean age of participants	
18–29 years	8 (23)
30–49 years	16 (46)
50–69 years	10 (29)
≥ 70 years	1 (3)
Percentage of women participants	
≤ 25%	3 (9)
26–50%	8 (23)
51–75%	15 (43)
76–100%	9 (26)
Study location	
Europe	20 (57)
North America	10 (29)
Other	3 (9)
Not reported	2 (6)
Year of publication	
2000–2009	5 (14)
2010–2019	17 (49)
2020–2025	13 (37)

**Table 2:** Summary of studies investigating the role of self-efficacy and motivation as moderators

Author(s), publication year, study location	Intervention study design, follow-up time	Outcome and its assessment	Psychosocial factor and its assessment	Study population and characteristics	Type of analyses and main findings
Bennett et al., 2007 [32] Location not stated	Two sessions of motivational interviews, via telephone with physical activity counselor. RCT with 6-month follow-up	Physical activity measured as weekly caloric expenditure based on self-reported physical activity assessed by the Community Healthy Activities Model Program for Seniors Physical Activity Questionnaire for Older Adults.	Self-efficacy for regular physical activity, measured by a questionnaire with good internal consistency.	Adult and physically inactive cancer survivors recruited using advertising and word of mouth. At baseline: *n* = 56, mean age 55.5 ± 8.9 (intervention group) 60.1 ± 11.0 (control group), 89% women. At follow up: *n* = 49 (13% attrition).	Moderator analysis: Interaction tested. A moderating effect was found. In the intervention group, those with high self-efficacy had a greater increase in physical activity, compared to those with low self-efficacy. No difference was found in the control group.
Benyamini et al., 2013 [33] Israel	Weight loss program focused on lifestyle changes. Quasi experimental study with 15-month follow-up	Body mass index (BMI) based on self-reported weight.	Ability to control eating, assessed by the Eating Self-efficacy Scale, with proven internal consistency and construct validity.	Adults taking part in weight loss groups. At baseline: *n* = 632, mean age 46 ± 11, 90% women. At follow up: *n* = 570 (10% attrition).	Moderator analysis: Interaction tested. No moderating effects were found.
Churchill et al., 2014 [34] UK	Reading of a temporal (day vs. year) framed message on the benefits of avoiding eating high calorie snacks. RCT with 1-week follow-up	Self-reported consumption of unhealthy snacks assessed by a single item open question.	Self-efficacy was assessed by the short form of the Weight Efficacy Lifestyle Questionnaire, with good internal consistency.	Convenience sample recruited from university staff. At baseline: *n* = 146, mean age 42.4 ± 11.3, 74% women. At follow up: *n* = 95 (65% attrition).	Moderator analysis: Interaction tested. A moderating effect was found. Self-efficacy was associated with lower consumptions of snacks in the year-framed message group, compared to the day-framed message group.
Elbert et al., 2017 [35] Netherlands	Online personalized health messages via a mobile phone app regarding fruit and veg intake. Quasi experimental study with 2- week follow-up	Self-reported intake of fruit and vegetable assessed by Food Frequency Questionnaire	Self-efficacy assessed by two items on perceived difficulty of eating sufficient fruit and vegetables, respectively. No information regarding reliability or validity.	Participants were students recruited from a pool of current and former psychology students at the local university. At baseline: *n* = 137, mean age 23.7 ± 7.0, 80% women. At follow up: *n* = 112 (18% attrition).	Moderator analysis: Interaction tested A moderating effect was found. In the intervention group, compared to controls, vegetable intake increased more for those with *low* self-efficacy.
Luszczynska et al., 2011 [36] *Substudy 1* Poland	In-person session focusing on goal setting and action planning. Cluster RCT with 4-week follow-up	Self-reported physical activity for the past 14 days.	Self-efficacy assessed by 5 items based on frequent barriers to behavior change, which have shown close to acceptable levels of internal consistency (alpha = 0.67).	Adult diabetics recruited via diabetes clinics. At baseline: *n* = 58, mean age 48.2 ± 17.9, 57% women. Attrition 0%	Moderator analysis: Interaction tested. A moderating effect was found. Those with high self-efficacy benefited from the intervention, whereas those with low self-efficacy did not.
Luszczynska et al., 2011 [36] *Substudy 2* Poland	Web-based action planning. RCT with 3-year follow-up	Self-reported vigorous physical activity.	Recovery self-efficacy assessed by four items (e.g.: “*I am able to start regular running again even if I did not run for some time because I felt weak*”) which have shown acceptable internal reliability.	Adults (16–60 years), regularly engaged in vigorous physical activity, recruited via advertisement on website on running. At baseline: *n* = 187, mean age 29.0 ± 9.3, 25% women. Attrition 31%%	Moderator analysis: Interaction tested. A moderating effect was found. Only those with high recovery self-efficacy in the intervention group managed to maintain their level of physical activity.
Pfeffer et al., 2019 [37] Location not stated	15 min face-to-face physical activity planning intervention. RCT with 1- week follow-up.	Self-reported amount of moderate-to-vigorous physical activity.	Strength of intention to perform moderate to vigorous physical activities > 30 min/day assessed by three items on unnamed questionnaire. No information on reliability and validity is provided.	Students recruited via online platform. At baseline: *n* = 116, mean age 22.7 ± 2.6, 67% women. At follow up: *n* = 107 (8% attrition).	Moderator analysis: Interaction tested. A moderating effect was found. Intention strength predicted physical activity only in the *control-group.*
Satia et al., 2001 [38] USA	Tailored self-help dietary intervention including a computer-generated personalized letter, behavioral feedback, motivational telephone call, and supplementary materials. RCT with 12-month follow-up.	Self-reported intake of fat, fruit, and vegetables. Fat intake assessed with a modified version of the Fat-related Diet Habits Questionnaire.	Intrinsic- and extrinsic motivation assessed by a modified version of a questionnaire on cancer risk behavior. Information on internal consistency suggests inadequate reliability.	Adults recruited from enrollees of a health maintenance organization. At baseline: *n* = 1205, mean age 45.8 ± 14.6, 50% women. Attrition not reported.	Moderator analysis: Interaction tested. A moderating effect was found. The association between motivation by self-image and reduced fat intake was stronger in the intervention group than among controls.
Shahab et al., 2011 [39] UK	Targeted feedback on participants carbon monoxide levels in the blood (measured in lab) in relation to the development of CVD. Single blinded RCT with 6-month follow-up.	Smoking habits assessed by self-reports via the single question “*Have you smoked within the past seven days?*”.	Self-efficacy assessed via two items: “*how confident are you to be able to stop smoking?*” and “H*ow easy will it be to stop smoking?*”. These items have demonstrated acceptable internal reliability.	Adults recruited via e-mail, newspaper advertisements, flyers, and posters. At baseline: *n* = 160, mean age 31.7 ± 10.7, 44% women. At follow up: *n* = 109, (32% attrition).	Moderator analysis: Interaction tested. A moderating effect was found. Higher self-efficacy was associated with a higher likelihood of smoking cessation in the intervention group but not in the control group.
Steptoe et al., 2000 a [40] UK	Brief behavioral counselling in primary care RCT with 12-month follow-up	Physical activity assessed by self-reported number of sessions of physical activity (> 20 min) during the past 4 weeks.	Self-efficacy for physical activity was assessed with 3-item scale, which has demonstrated acceptable internal consistency. Motivation to change assessed with single item measure. No information on reliability and validity is provided.	Convenience sample with patients with raised risk of CVD, recruited during general practitioner consultations, leaflets etc. At baseline: *n* = 883, mean age 49.1 ± 11.2, 54% women. At 12 months follow-up: *n* = 418 (47% attrition).	Moderator analysis: Interaction tested. No moderating effects were found.
Stewart-Knox et al., 2022 [41] Greece, Germany, Ireland, Nether-lands, Poland, Spain, UK,	Personalized dietary advice. RCT with 6-month follow-up	Healthy diet was assessed by self-reports via the Healthy Eating Index and the Mediterranean Diet Index.	Nutrition self-efficacy was measured by the Perceived Self-Efficacy Scale, which has demonstrated good internal consistency.	Adult volunteers. Recruitment strategy not reported. At baseline: *n* = 1607, mean age 40.0 ± 13, 58% women. At follow up: *n* = 763 (53% attrition).	Moderator analysis: Interaction tested. No moderating effects were found.

*Interaction tested* means that an interaction between group belonging (e.g. intervention vs. control) and a psychosocial factor on the outcome was included in a model

**Table 3:** Summary of studies investigating the role of cognitive factors as moderators

Author(s), publication year, study location	Intervention study design, follow-up time	Outcome and its assessment	Psychosocial factor and its assessment	Study population and characteristics	Type of analyses and main findings
Bickmore et al., 2013 [62] USA	Daily interactions with a computer-animated virtual exercise coach. Single blinded RCT with 12-month follow-up.	Physical activity measured as daily step count assessed by pedometer.	Health literacy assessed by Test of Functional Health Literacy in Adults. No information on reliability or validity is provided.	Older physically inactive adults, recruited from outpatient clinics. At baseline: *n* = 263, mean age 71.3 ± 5.4, 61% women. At follow up: *n* = 128, (49% attrition)	Moderator analysis: Subgroup analysis^a^ performed without testing for an interaction^b^ A moderating effect was found. In the intervention group, but not among controls, those with adequate health literacy walked more after the intervention compared to those with inadequate health literacy.
Elbert et al., 2016 [63] Netherlands	Mobile phone app that communicated health information either through text or auditory methods RCT with 6-month follow-up	Fruit and vegetable intake assessed by a Food Frequency Questionnaire	Health literacy assessed by three items with good internal consistency. Perceived health assessed by a non-validated single item where participants were asked to rank their health on a 6-point scale.	Adults (≥ 16 years) recruited via various advertisement campaigns. At baseline: 342, mean age 41.4 ± 14.6, 73% women. At follow up: 146 (55% attrition).	Moderator analysis: Interaction tested A moderating effect was found. High health literacy and low perceived health predicted a greater effect of the intervention.
Harris et al., 2014 [64] UK	Screen-based health messages describing benefits of eating fruit and vegetables, augmented by information about the implications for heart disease RCT with 2 × 2 design, with 3-month follow-up.	Fruit and vegetable intake assessed by 3 self-report measures to determine intake during (1) a typical day; (2) the previous 24 h; (3) a typical week.	Self-affirmation and non-affirmation were induced by asking participants why their most important value was important to them. Non-affirmation was induced by asking the participants why their least important value may be important to someone else.	Staff and students from Sheffield University, recruited via university e-mail lists. At baseline: *n* = 332, mean age 22.3 ± 5.9, 72% women. At follow-up: *n* = 162 (51% attrition).	Moderator analysis: Interaction tested A moderating effect was found. Self-affirmed participants had higher fruit and vegetable intake compared to non-affirmed participants.
Webb Hooper et al., 2013 [65] USA	Placebo tailored self-help booklets on smoking cessation and relapse prevention RCT with 6-month follow-up	Smoking cessation measured by self-reported 7-day point prevalence abstinence. Smoking abstinence confirmed by breath carbon monoxide detectors at follow-up.	Cognitive processing style assessed by an adapted version of the Media Information Processing Measures in Surveys scale, which demonstrated good internal consistency.	Current smokers of at least 5 cigarettes per day, recruited via advertisements. At baseline *n* = 424, mean age 41.76 ± 11.31, 57% women. At follow-up: *n* = 309 (27% attrition).	Moderator analysis: Interaction tested A moderating effect was found. Higher heuristic cognitive processing was associated with higher likelihood of smoking cessation

*Interaction tested* means that an interaction between group belonging (e.g. intervention vs. control) and a psychosocial factor on the outcome was included in a model

**Table 4:** Summary of studies investigating the role of social support and relationship quality as moderators

Author(s), publication year, study location	Intervention study design, follow-up time	Outcome and its assessment	Psychosocial factor and its assessment	Study population and characteristics	Type of analyses and main findings
Aggarwal et al., 2010 [42] USA	Lifestyle intervention with personalized assessment of CVD-risk, and diet and lifestyle counselling Prospective sub-study of an RCT with 12-month follow-up.	Diet was assessed with the National Heart, Lung, and Blood Institute’s Eggs, Dairy, Fried foods, In baked goods, Convenience foods, Table fats Snacks assessment tool. Scores > 70 indicate non-adherence to healthy diets.	Social support was measured using the Enhancing Recovery in Coronary Heart Disease Patients Social Support Instrument, which has demonstrated good reliability and validity.	Blood relatives or cohabitants of hospitalized cardiac patients. At baseline: *n* = 501, mean age 49 ± 13, 66% women. At follow-up: *n* = 468 (6% attrition).	Moderator analysis: Interaction tested. No moderating effect was found.
Keller et al., 2020 [43] Germany	Dyadic planning to change health relevant behavior. RCT with 1-year follow-up. Data from this trial is also used by Knoll et al. (2017).	Physical activity assessed using accelerometer worn on four occasions during the study.	Relationship quality assessed using the German 12-item short form of the Dyadic Adjustment Scale, which has adequate levels of internal consistency.	Participants were healthy, physically active, adult couples recruited through advertising. At baseline: *n* = 269 couples, mean age 37.7 ± 15.6, 49% women. Attrition n/a.	Moderator analysis: Interaction tested. A moderating effect was found. Those with lower relationship quality showed stable physical activity levels while physical activity of those with higher relationship quality increased significantly over time.
Knoll et al., 2017 [44] Germany	Dyadic and individual planning to change health relevant behavior. RCT with 6-week follow-up, with either dyadic planning or individual planning. Data from this trial is also used by Keller et al. (2020).	Physical activity assessed using accelerometer measuring minutes of moderate to vigorous physical activity.	Relationship quality assessed using the German 12-item short form of the Dyadic Adjustment Scale, which has adequate levels of internal consistency.	Participants were healthy adult couples recruited through advertising. At baseline: *n* = 346 couples, mean age 37.7 ± 5.6, 50% women. At follow-up: *n* = 331 couples (4% attrition)	Moderator analysis: Interaction tested. A moderating effect was found. Low relationship quality was associated with *increased* physical activity in individual planners and *decreased* physical activity in dyadic planners. High relationship quality was associated with decreased physical activity in individual planners, and with no change in dyadic planners.
Lau-Barraco et al., 2022 [45] USA	Personalized feedback regarding alcohol use and its consequences. RCT with 3-month follow-up	Alcohol consumption assessed with self-report using The Daily Drinking Questionnaire.	Social network was assessed with a modified version of the Social Network Map. No information on reliability or validity is reported.	Heavy drinkers, aged 18–25, recruited through advertisements. At baseline: *n* = 164, mean age 22.0 ± 2.0, 34% women. Attrition not reported.	Moderator analysis: Interaction tested. A moderating effect was found. Participants with more stable social networks reduced their drinking more than those with less stable networks.
Lawler et al., 2014 [46] Australia	12-month telephone counselling intervention for physical activity and diet Cluster RCT with 18-month follow-up	Physical activity assessed in the Active Australia Survey. Fruit and vegetable intake measured using two items from the Australian National Nutrition Survey. Fat and fiber intake assessed with Food Frequency Questionnaire.	Social support for physical activity and healthy eating was assessed with a modified version of the Chronic Illness Resource Survey. No information on reliability or validity is reported.	Type 2 diabetes and/or hypertension patients, recruited through primary care clinics. At baseline: *N* = 434, Mean age 58.7 ± 11.7. 61% women. At follow-up: *n* = 306 (29% attrition).	Moderator analysis: Interaction tested. A moderating effect was found. In the control group, but not in the intervention group, high social support was related to greater improvements in physical activity.
Steptoe et al., 2000 a [40] UK	Brief behavioral counselling. RCT with 12-month follow-up.	Physical activity assessed by self-reported number of sessions of physical activity (> 20 min) during the past 4 weeks.	Social support assessed by questionnaire, the name and validity of which was not reported. Social support for physical activity assessed by the single item “*Does the person most important to you encourage you to eat a low-fat diet?“.*	Patients with at least one CVD risk factor, recruited from general practices. At baseline: *n* = 883, mean age 49.1 ± 11.2, 5% women. At follow-up: *n* = 418 (47% attrition).	Moderator analysis: Interaction tested. A moderating effect was found. In the intervention group, but not in controls, higher social support predicted increased physical activity.
Steptoe et al., 2000 b [47] UK	Behavioral counselling vs. general health promotion advice. RCT with 4-month follow-up.	Fat intake assessed using Dietary Instrument for Nutrition Education	Social support, from family from others, assessed by 6 questions, name and validity of questionnaire not reported. Social support for physical activity assessed by the single item “*Does the person most important to you encourage you to eat a low-fat diet?”*	Patients with at least one CVD risk factor, recruited from general practices. At baseline *n* = 365, mean age 52.1 ± 10.1, 51% women. At follow up: *n* = 291 (20% attrition).	Moderator analysis: Interaction tested. A moderating effect was found. In the control group, but not the intervention group, those with higher support from family had greater fat reduction.
Westland et al., 2020 [48] Netherlands	Nurse-led behavioral change consultations. Cluster RCT with 6-month follow-up.	Physical activity measured by accelerometers and activity log.	Social support assessed with the Multidimensional Scale of Perceived Social Support. No information on reliability or validity is reported.	Sedentary adults (40–75 years) at risk for CVD recruited through general practices. At baseline *n* = 195 (IG: *n* = 93; CG: *n* = 102). IG mean age 61.9 ± 9.1, 41% women. CG mean age 63.4 ± 8.3, 35% women. The intervention group at follow up: *n* = 70 (25% attrition).	Moderator analysis: Interaction tested. A moderating effect was found. Patients with *low* social support had the greatest increase in physical activity.

Note: “*Interaction tested*” means that an interaction between group belonging (e.g. intervention vs. control) and a psychosocial factor on the outcome was included in a model

**Table 5:** Summary of studies investigating the role of mental health and stress as moderators

Author(s), publication year, study location	Intervention study design, follow-up time	Outcome and its assessment	Psychosocial factor and its assessment	Study population and characteristics	Type of analyses and main findings
Gaume et al., 2023 [49] Switzerland	Brief motivational Interviewing. RCT with 12-month follow-up	Self-reported number of heavy drinking days, assessed by a 30-day timeline follow-back.	Depression and Anxiety assessed by the Patient Health Questionnaire-4, with adequate levels of internal consistency. Importance and confidence to change assessed by the Readiness Rulers Questionnaire. No information on reliability or validity was provided. Trait reactance assessed by the Hong Psychological Reactance Scale, with adequate level of internal consistency.	Young adults (18–35 years) seeking urgent care for intoxication. At baseline: *n* = 344, mean age 24.2 ± 4.7, 75% women. Attrition n/a.	Moderator analysis: Interaction tested. A moderating effect was found. After latent class analysis, the profile with the greatest intervention effect included persons with low anxiety and depression as well as low confidence, high importance to change score, and high severity of alcohol use
Goessl et al., 2021 [50] USA	Weight loss program given individually in-person, in groups in-person, or in groups via conference calls. Cluster RCT with 2-year follow-up.	Physical activity assessed by the Past Week Modified Activity Questionnaire. Diet, i.e. fruit and vegetable intake, assessed by the National Cancer Institute’s Quick Food Scan and Fruit and Veg Screener. Weight assessed with calibrated scales in clinic	Affective disorder was assessed by the following three non-validated items “*Are you currently in counseling or therapy for depression?*”, “*Are you currently in counseling or therapy for anxiety?*” and “*Do you currently take medications for adjustment issues*,* depression*,* anxiety*,* or nerves?”*	Rural obese adults (20–75 years) recruited via primary care practices. At baseline *n* = 1407, mean age 54.7 ± 11.5, 77% women. At follow-up: *n* = 1220 (13% attrition).	Moderator analysis: Subgroup analysis performed without testing for an interaction. A moderating effect was found. Those with an affective disorder had less weight loss, less improvement in physical activity and had lower fruit and vegetable intake.
Kekkonen et al., 2025 [51] 10.1186/s12877-025-05830-y Finland	Multidomain lifestyle intervention with individually tailored exercise session and dietary counselling RCT with 2-year follow-up	Physical activity assessed by self-reported frequency of exercise activities per week	Depression assessed by the validated Zung Self-Rating Depression Scale. Mild depression is defined as a score of ≥ 40.	60–77-year-olds without major depression and at risk for cognitive decline, recruited from earlier population-based observational surveys. At baseline: *n* = 1259. In the intervention group, mean age 68.9 ± 4.6, 44.3% women. In the control group, mean age 68.5 ± 4.7, 47.8% women. At follow up: *n* = 1109 (12% attrition).	Moderator analysis: Interaction tested No moderating effects were found.
Neuvonen et al., 2022 [52] Finland	Multidomain lifestyle intervention with individually tailored exercise session and dietary counselling RCT with 2-year follow-up	Combined lifestyle score that included: Physical activity assessed by self-reported frequency of sports activities per week Adherence to national dietary recommendations assessed by 3-day food record 10-year CVD risk based on the FINRISK risk score Cognitive and social activities were assessed by the total amount spent per week doing a selection of activities e.g. reading and solving crosswords.	Depression assessed by the validated Zung Self-Rating Depression Scale, and self-reports on depressive symptoms and antidepressant medication from the FINRISK questionnaire. Health-Related Quality of Life assessed by the previously validated RAND-36 questionnaire.	60–77-year-olds without major depression and at risk for cognitive decline, recruited from earlier population-based observational surveys. At baseline: *n* = 1259. In the intervention group, mean age 69.5 ± 4.7, 45% women. In the control group, mean age 69.2 ± 4.7, 18% women. At follow up: *n* = 891 (29% attrition).	Moderator analysis: Interaction tested No moderating effects were found.
Pomp et al., 2013 [53] Germany	Computer-based self-regulation intervention to promote exercise Quasi experiment, with 6-week follow-up.	Physical activity (time spent per week) assessed by the Godin Leisure-Time Exercise Questionnaire.	Depression assessed with the 2-item version of the Patient Health Questionnaire-2, which has demonstrated good validity.	Individuals recruited from orthopedic rehabilitation clinics prior to the start of rehabilitation. At baseline: *n* = 581, mean age 48.4 ± 10, 86% women. At follow up: *n* = 361, (37% attrition)	Moderator analysis: Interaction tested. No moderating effects were found.
Trief et al., 2014 [54] USA	Phone-based intervention given individually or in group. Program included goal setting, diet- and physical activity modification, and problem solving. RCT with 24-month follow-up.	Weight measured by research nurse at primary care practices	Depression assessed by the Center for Epidemiologic Studies-Depression Scale which has good internal consistency. Stress assessed by the Perceived Stress Scale, which has been validated.	Adults (> 18 years) with BMI ≥ 30 and metabolic syndrome, recruited via primary care practices. At baseline: *n* = 257. Individual arm: mean age 50.7 ± 13.1, 78% women. Group arm: mean age 52.7 ± 12.8, 72% women. At follow-up: *n* = 135 (48% attrition)	Moderator analysis: Subgroup analysis performed without testing for an interaction. A moderating effect was found. Those with more depressive symptoms showed less weight loss at 6 and 12 months and those with higher stress had less weight loss at 6, 12 and 24 months.
van Dammen et al., 2021 [55] Netherlands	Individualized behavioral modification counselling. RCT with 6-years follow-up	Weight measured by nurses.	Childhood adversity assessed by the Life Events Checklist 5 Questionnaire, which has demonstrated acceptable psychometric properties.	Infertile women (18–39 years) with BMI ≥ 29, enrolled in an ongoing trial. At baseline: *n* = 110, mean age 30.3 ± 4.1, 100% women. Attrition n/a.	Moderator analysis: Interaction tested A moderating effect was found. Those with experiences of childhood adversity were more likely to lose weight compared to women without childhood adversity.

*Interaction tested* means that an interaction between group belonging (e.g. intervention vs. control) and a psychosocial factor on the outcome was included in a model. *“Subgroup analysis”* means that the change in outcome *only in the intervention arm* was evaluated across subgroups

**Table 6:** Summary of studies investigating the role of personality and emotions as moderators

Author(s), publication year, study location	Intervention study design, follow-up time	Outcome and its assessment	Psychosocial factor and its assessment	Study population and characteristics	Type of analyses and main findings
Bryan et al., 2013 [56] USA	Print-based physical activity lifestyle intervention, education, and individually tailored messages. RCT with 12-month follow-up.	Physical activity. Intensity and frequency assessed through self-report with the 7-Day Physical Activity Recall Questionnaire.	Experienced affect during exercise was assessed by self-report with the Feeling scale, a single item (exact wording not reported) 11-point measure assessed the valence component of affect. No information on reliability or validity is reported.	Sedentary adults (18–45 years) recruited from the general population. At baseline: *n* = 238, mean age 28.2 ± 7.95, 80.4% women. At follow-up *n* = 219 (8% attrition).	Moderator analysis: Interaction tested^a^ No moderating effects were found.
Burnos et al., 2021 [57] Poland	Lifestyle intervention for diet and physical activity with motivational interviewing. Longitudinal study with 15-month follow-up without control group.	BMI and health behaviors including eating habits and physical activity assessed with the Inventory of Health behavior.	Personality traits were measured with the use of NEO Five Factor Inventory, which is a valid and reliable instrument. Temperament traits were assessed with the Formal Characteristics of behavior– Temperament Inventory, which has good reliability and validity.	Adult patients with metabolic syndrome recruited from a hospital. At baseline: *n* = 80, mean age 45.3 ± 9.8. 22% women. At follow-up: *n* = 50 (38% attrition)	Moderator analysis: Subgroup analysis^b^ performed without testing for an interaction A moderating effect was found. Cluster analysis showed that those high in neuroticism, perseveration, and emotional reactivity had a greater increase in physical activity compared to other categories. No moderating effect was found for diet or BMI.
Capone et al., 2009 [58] USA	Brief motivational intervention and alcohol expectancy challenge RCT with 2 × 2 factorial design, and 6-months follow-up	Alcohol consumption was measured by the timeline follow back. Alcohol-related problems were assessed with the Young Adult Problems Screening Test.	Need for cognition was assessed with the Need for Cognition Scale, which has good psychometric properties. Impulsivity and sensation seeking was assessed with the Impulsive Sensation Seeking Scale from Zuckerman and Kuhlman’s Personality Questionnaire, which has shown adequate reliability.	Heavy drinking college students recruited via advertisements At baseline *n* = 335, mean age 20.9 ± 0.9, 53% women. At follow-up *n* = 243 (28% attrition).	Moderator analysis: Interaction tested A moderating effect was found for need for cognition, but not for impulsivity and sensation seeking. Those with high need for cognition showed greater reduction in alcohol consumption.
Gómez-Martínez et al., 2022 [59] Spain	Lifestyle intervention, including motivational interviewing. Observational prospective cohort study with 3-year follow-up.	Diet assessed with a food frequency questionnaire.	Impulsivity assessed with the Impulsive Behavior Scale, validated for Spanish populations.	Older adults with overweight or obesity recruited via primary care health centers. At baseline *n* = 487, mean age 65.3 ± 4.7, 52% women. At follow up *n* = 462 (5% attrition)	Moderator analysis: Interaction tested A moderating effect was found. Higher impulsivity was associated with lower adherence to healthy diet in the intervention group, but not in the control group.
Sakane et al., 2023 [60] Japan	Smart phone app for self-help guidance to prevent CVD, diabetes in middle-aged. RCT with 12-week follow-up	Weight reported via a Bluetooth scale.	Personality was categorized into four types: Challenger (self-realization and a sense of growth; fact-based extrovert), Entertainer (connection and gratitude; relationship introvert), Communicator (optimism; relationship introvert) and Walker (do things at my own pace; fact-based introvert). No information is provided on how personality traits were assessed.	Men aged 40–64 years with BMI > = 25 with hypertension, recruited through recruitment site for product development and research. At baseline *n* = 78, mean age 52 ± 6.5, 0% women. At follow-up: *n* = 74 (5% attrition).	Moderator analysis: Subgroup analysis performed without testing for an interaction A moderating effect was found. Participants categorized as “*Walkers*” had greater weight loss than those categorized as “*Challengers*”.
Stieger et al., 2023 [61] USA	Digital self-control intervention to increase physical activity using a coaching app. RCT with 12-week follow-up.	Physical activity assessed by the International Physical Activity Questionnaire. Activity trackers objectively assessed the number of daily steps and sessions of moderate-to-vigorous physical activity.	Conscientiousness was assessed with the Big-five Inventory-2, which has good validity and reliability.	Middle-aged adults recruited by advertisements. At baseline *n* = 86, mean age 46.96 ± 9.54, 78% women. At follow-up: *n* = 81 (attrition: 6%).	Moderator analysis: Interaction tested A moderating effect was found. Participants in the intervention group with high, compared to low, conscientiousness had greater increase in daily steps.

“*Interaction tested*” means that an interaction between group belonging (e.g. intervention vs. control) and a psychosocial factor on the outcome was included in a model. *“Subgroup analysis”* means that the change in outcome *only in the intervention arm* was evaluated across subgroups

Overall, 27 (77%) of the included studies found a moderating effect of a psychosocial factor, whereas the remaining 8 did not. The studies were categorized based on the type of psychosocial factor investigated. Among the studies included in the category “*cognition*”, all studies reported moderating effects, and for the categories ”*social support and relationship quality*” and “*personality and emotions*” one study in each category failed to find a moderating effect. Results were more varied in the other categories. In the “*motivation and self-efficacy*” category, three out of eleven studies did not find a moderating effect, and in the “*mental health*” category, three out of six studies reported no moderating effects.

The included studies cover a wide range of psychosocial factors, which were not always assessed by validated instruments, with self-efficacy being the most frequently assessed psychosocial factor. Notably many studies focused on non-modifiable factors. In addition, studies were found to be heterogenous with regards to e.g., sample characteristics, type of lifestyle interventions, and outcome measures. Regarding outcomes, most studies focused on physical activity, diet or body weight, whereas only a few focused on alcohol and smoking. In Tables 2, 3, 4, 5, 6 the results for each moderator category are summarized and reported in more detail.

### Studies investigating self-efficacy and motivation as moderators

Eleven studies [32–41] described in more detail in Table 2, investigated whether motivation or self-efficacy moderated the effects of interventions aimed at physical activity (*n* = 5), diet (*n* = 4), weight loss (*n* = 1), or smoking cessation (*n* = 1). Eight studies drew data from RCTs. Sample size was generally high, with only Bennet et al. [32] and sub-study 1 of Luszczynska et al. [36] reporting a sample size < 100 participants. Three studies investigated samples with a mean age < 30 years. In five of the studies, the gender distribution was roughly equal (40–60% women), five studies had samples with clear female majority (> 60% women), and one had a clear male majority (25% women). In seven studies, self-efficacy or motivation were assessed by up to three single items that were not adapted from any validated instrument.

The results indicate that self-efficacy can moderate intervention effects, though the results are not conclusive. Three out of the nine studies investigating self-efficacy reported a moderating effect on the outcome. In addition, Satia et al. [38] found that motivation by self-image moderated the effect of a dietary intervention, and Pfeffer et al. [37] found that intention strength moderated the effect of a short planning intervention targeting physical activity.

### Studies investigating social support and relationship quality as moderators

As seen in Table 3, eight studies [40, 42–48] investigated social support and relationship quality as moderators of the effect of interventions aimed at improving physical activity (*n* = 5) and diet (*n* = 3). One study used alcohol consumption as an outcome [45] and this study was also the only one with a sample with a mean age < 30 years. Overall, sample sizes were high, ranging from 164 to 883. As for gender distribution, half (*n* = 4) of the studies had samples with roughly equal gender distribution (40–60% women), two studies had a clear female majority and two a clear male majority (i.e., > 60% men or women, respectively). Six studies used an RCT design, two were cluster RCT’s, and one was a prospective study [42] drawing data from an RCT. Of note is that the studies of Knoll et al. [44] and Keller et al. [43] used data from the same trial with participants who were physically active. Seven out of the eight studies reported that social support or relationship quality acted as moderators (*n* = 7) of the intervention effect, the only exception being the study by Aggarwal et al. [42]. However, only five of the studies evaluated social support or relationship quality using validated instruments.

### Studies investigating mental health and perceived stress as moderators

Seven studies [49–55] (see Table 4) investigated the effects of mental health factors as moderators of interventions aimed at physical activity (*n* = 3), weight loss (*n* = 3), diet (*n* = 1), alcohol consumption (*n* = 1), or a combined CVD risk assessment (*n* = 1). Overall, sample sizes were large, ranging from 110 to 1407 participants. Two studies investigated samples with a mean age ≤ 30 years. Five studies had a clear female majority (> 60% women) and in the study by Neuvonen et al. [52] the gender distribution differed greatly between the intervention group (45% women) and control group (18% women). One study used a quasi-experimental design in which non-randomized participants were included in the control group, another was a cluster RCT, and all other studies (*n* = 5) were RCTs. The measurement of mental health and stress varied considerably across studies; however, six of the studies used validated instruments to assess mental health or perceived stress.

Four of the seven studies reported that mental health or perceived stress moderated the effect of lifestyle interventions. For example, weight loss interventions were found to be more successful in those with lower levels of depression, anxiety, and stress [50, 54] and amongst women with adverse childhood experiences [55]. In two studies with varied follow-up, one only six weeks [53] and the other 2 years [51], depression was not a significant moderator of the intervention effect on physical activity in a study with only six weeks of follow-up. Nor did depression moderate the effect of a multidomain lifestyle intervention on a composite lifestyle score that included physical activity, diet, CVD risk score, amongst others [52]. With regards to alcohol consumption, a brief motivational interview intervention had the largest effect on those with lower anxiety and depression [49].

### Studies investigating personality and emotions as moderators

Six studies [56–61] investigated aspects of personality and emotion as moderators of intervention effects regarding diet (*n* = 2), physical activity (*n* = 3), weight (*n* = 2), and alcohol consumption (*n* = 1) (see Table 5). Two studies had a quasi-experimental design, and three were RCTs. The sample sizes varied, and three studies used small samples of < 100. In four studies, participants were middle-aged. One study included college students [58], and one study included older adults [59]. In two studies, the samples included only women or mostly women, whereas two studies had an even gender distribution. In two studies, the samples included only men or mostly men. Regarding the assessment of moderators, four studies used psychometrically validated instruments, and in two studies, no information on reliability or validity was provided.

Five studies showed that aspects of personality and emotion had a moderating effect on the outcome in these lifestyle interventions, whereas one did not. The studies on physical activity indicated that higher conscientiousness [61] as well as higher neuroticism, perseveration and emotional reactivity [57] were related to a greater increase in physical activity, whereas the subjective response to physical activity in terms of affect did not moderate the intervention effect [56]. However, the reliability and validity of the moderator might be questioned as this study used a single-item measure. Personality traits were also related to intervention effects in terms of weight loss [60], as individuals who were classified as introverts (termed “*Walkers*” in the study) had greater weight loss than extroverts (termed “*Challengers*”). However, in this study, the assessment of personality and its reliability and validity was not described. With respect to healthy diet, there was a moderating effect of impulsivity, where high impulsivity was related to lower adherence [59].

### Studies investigating cognitive factors as moderators

Four studies [62–65] investigated cognitive factors as moderators of the effects of interventions aimed at physical activity (*n* = 1), diet (*n* = 2), and smoking cessation (*n* = 1). These studies are summarized in Table 6. Sample sizes ranged between 263 and 424 participants. Only one study [64] had a sample with a mean age < 30 years. Of the four studies, three had samples consisting of > 60% women. Webb Hooper et al. [65] relied on a validated instrument to assess the cognitive factor, whereas Bickmore et al. [62] did not. Elbert et al. [63] used a validated instrument in the assessment of health literacy, but not perceived health. Finally, in Harris et al. [64] self-affirmation was not assessed, but rather induced through manipulation.

Two studies that included diet as an outcome reported that health literacy [63] and self-affirmation [64], moderated the effect of the intervention on fruit and vegetable intake. The study that assessed physical activity showed that health literacy moderated the intervention’s effect on walking, which was objectively measured [62]. In the only study that assessed a smoking cessation intervention [65], the results showed that the intervention was more successful in those with higher heuristic cognitive processing.

### Description of types of intervention

An overview of the components included in the various interventions can be found in Table 7. Goal setting and planning was the most commonly used type of intervention (*n* = 24). Twelve studies used an intervention with only one component (health education, *n* = 4; behavioral counselling, *n* = 1; goal setting and planning, *n* = 7). Ten of the studies used interventions with three or more components. Overall, no clear pattern was observed in whether a moderation effect was more likely with certain types of interventions or not.

**Table 7:** Summary of psychosocial factors as moderators of behavior change and components of the intervention

First author and year of publication		Outcome category	Psychosocial factor	Moderating effect		Components of the intervention				
						Health education	Diet or exercise programme	Behavioral counselling	Goal setting and planning	Feedback and monitoring
Motivation and self-efficacy	Bennett, 2007 [32]	Physical activity	Self-efficacy		Yes			X	X	
	Benyamini, 2013 [33]	Weight	Self-efficacy		No				X	
	Churchill, 2014 [34]	Diet	Self-efficacy		Yes	X				
	Elbert, 2017 [35]	Diet	Self-efficacy		Yes	X				
	Luszczynska 2011 [36] Substudy 1	Physical activity	Self-efficacy		Yes				X	
	Luszczynska 2011 [36] Substudy 2	Physical activity	Self-efficacy		Yes				X	
	Pfeffer, 2019 [37]	Physical activity	Intention strength		Yes				X	
	Satia, 2001 [38]	Diet	Intrinsic and extrinsic motivation		Yes	X				X
	Shahab, 2011 [39]	Smoking	Self-efficacy		Yes	X				X
	Steptoe, 2000a [40]	Physical activity	Self-efficacy, motivation to change		No	X		X		
	Stewart-Knox, 2022 [41]	Diet	Self-efficacy		No	X				
Social support and relationship quality	Aggarwal, 2010 [42]	Diet	Social support		No	X		X	X	
	Keller, 2020 [43]	Physical activity	Relationship quality		Yes				X	
	Knoll, 2017 [44]	Physical activity	Relationship quality		Yes				X	
	Lau-Barraco, 2022 [45]	Alcohol consumption	Social network		Yes			X		X
	Lawler, 2014 [46]	Physical activity, diet	Social support		Yes	X		X	X	X
	Steptoe, 2000a [40]	Physical activity	Social support		Yes	X		X		
	Steptoe, 2000b [47]	Diet	Social support		Yes	X		X	X	
	Westland, 2020 [48]	Physical activity	Social support		Yes	X		X	X	X
Mental health	Gaume, 2023 [49]	Alcohol consumption	Depression, anxiety confidence		Yes			X	X	
	Goessl, 2021 [50]	Weight, diet, physical activity	Depression, anxiety, adjustment issues		Yes		X	X	X	X
	Kekkonen, 2025 [51]	Physical activity	Depression		No		X	X	X	X
	Neuvonen, 2022 [52]	Diet, Physical activity, Lifestyle score	Depression, HRQOL		No		X	X	X	X
	Pomp, 2013 [53]	Physical activity	Depression		No				X	X
	Trief, 2014 [54]	Weight	Perceived stress Depression,		Yes				X	X
	Van Dammen, 2021 [55]	Weight	Childhood adversity		Yes			X	X	X
Personality and emotions	Bryan,2013 [56]	Physical activity	Affect during exercise		No	X			X	X
	Burnos, 2021 [57]	Physical activity	Personality traits		Yes	X		X		
	Capone, 2009 [58]	Alcohol consumption	Need for cognition, impulsivity, sensation seeking		Yes			X		
	Gómez-Martínez, 2022 [59]	Diet	Impulsivity		Yes	X		X		
	Sakane, 2023 [60]	Weight	Personality traits		Yes	X			X	X
	Stieger, 2023 [61]	Physical activity	Conscientiousness		Yes				X	
Cognition	Bickmore, 2014 [62]	Physical activity	Health literacy		Yes				X	X
	Elbert, 2016 [63]	Diet	Health literacy, perceived health		Yes			X	X	X
	Harris, 2014 [64]	Diet	Self-affirmation		Yes	X			X	
	Webb Hooper, 2013 [65]	Smoking	Cognitive processing style		Yes	X				

## Discussion

The aim of this scoping review was to systematically chart studies assessing psychosocial factors as moderators of behavioral change in primary CVD prevention targeting adult populations. The further aim was to summarize current knowledge and identify potential knowledge gaps.

The overall findings from this review indicate that a wide range of psychosocial factors may be of importance in lifestyle interventions, and may therefore have implications for developing or improving lifestyle interventions in the future. However, it is difficult to draw more detailed conclusions due to the heterogeneity among the studies.

A total of 35 studies were included. The most common lifestyle behaviors targeted with interventions were physical activity, followed by diet, whereas fewer studies evaluated the intervention effects on alcohol consumption and smoking cessation. Only one study evaluated a composite CVD risk score as an outcome. Overall, most studies reported moderating effects of a psychosocial factor on the outcome in the intervention group. Other studies reported a moderating effect only in the control group, which can still indicate the relevance of the psychosocial factor in relation to the intervention. For example, in Pfeffer et al. (2019), high intention strength predicted increased physical activity only in the control group, which can be interpreted as the intervention helping those with low intention strength to overcome this deficit. We charted the components of lifestyle interventions present in the different studies (Table 7), but given the heterogeneity among the studies, no visible patterns were found between specific types of interventions and the moderating effect of the psychosocial factors.

### Evidence within categories of psychosocial moderators

The studies on self-efficacy and motivation show mixed results. This is in line with the results of a review on factors associated with attendance and attrition in weight management interventions [18] which also showed inconsistent results for self-efficacy. Most studies in this category showed a moderating effect such that higher self-efficacy or motivation was associated with larger intervention effects. In contrast, Elbert et al. (2017) [35] showed that those with low self-efficacy benefited more from an intervention aiming to increase vegetable consumption among university students.

Of the eight studies investigating social support and relationship quality, seven reported a moderating effect. However, in Westland et al. [48], *low* social support was associated with *increased* physical activity in the intervention group. Most of the studies included in this review investigated samples with a majority of women, however, the studies investigating the role of social support and relationship quality were exceptions in this regard, as four of the studies in this category had roughly equal gender distribution. Overall, the results are in line with previous qualitative studies that have identified social support as a perceived facilitator of adherence to lifestyle modification programs [22] and of the maintenance of changed health behaviors over time [21]. These results are also in line with a 2017 review by Leung et al. [18] which concluded that social support is associated with a higher level of attendance in weight management interventions.

Overall, the studies investigating the moderating effect of mental health indicate that aspects of poor mental health (e.g., depression, anxiety, and perceived stress) are associated with less favorable outcomes in lifestyle interventions. In two studies in which no moderating effects were found, the prevalence of depression among participants was low. It is possible that those with better mental health also have higher self-efficacy, motivation and restraint behavior, factors that have been associated with weight loss [66]. In addition, those with higher levels of perceived stress may be less likely to regulate their eating behavior [67]. Depression, when studied in isolation, did not moderate the effect of an intervention aimed at changing lifestyle behaviors [52, 53], but this may only apply to people with less severe depression. In one of these studies [52] those with major depression were excluded and in the other study [53] the prevalence of depression was low. Furthermore, people with depression can be less likely to participate in and complete a lifestyle intervention program [20]. It is therefore difficult to determine whether depression is a moderator of the success of a lifestyle intervention when those at the higher end of the depression spectrum are not included in studies. Comparatively, a study that included a higher proportion of people with depression, half of whom were on treatment found a significant moderating effect [54].

Taken together, the studies on personality and emotion indicate positive moderating effects. More specifically, the personality traits neuroticism, perseveration, and conscientiousness as well as optimism and self-compassion were positively related to intervention effects, whereas impulsivity was inversely related to intervention effects. Previous research on the association between personality and health behavior in general provides support for these findings. For example, studies indicate that conscientiousness is positively related to health behaviors [68], such as healthy eating [69, 70] and physical activity, and is negatively related to sedentary behavior [71]. With regard to neuroticism and emotional instability, some previous studies indicate that these constructs are negatively related to health behavior, but the findings are mixed [72]. Recent research suggests that “*healthy neuroticism*”, defined as the interaction of neuroticism and conscientiousness, is positively linked to health-related behaviors [64]. This is in line with the findings reported in Burnos et al. [57] in which participants high in neuroticism, perseveration (a facet of conscientiousness) and emotional reactivity improved in terms of health practices. As for the personality traits of introversion and extraversion, the findings of Sakane et al. [60] indicate that introvert participants have a larger intervention effect in terms of weight loss compared to more extrovert participants. This is in line with the findings of Sutin et al. [71], reporting that extraversion was positively associated with BMI in a large community sample. However, other studies have reported none [73, 74] or negative [75] associations between weight and extraversion.

Regarding studies on cognition, all studies indicated moderating effects of cognitive factors, more specifically health literacy, self-affirmation and cognitive processing style. This review included two studies that showed that higher health literacy enhanced the intervention effect to improve physical activity and dietary behaviors [62, 63]. The literature indicates that health literacy also may enhance self-efficacy and perception of risk [76], which may further contribute to behavior change in lifestyle interventions. Further, those who perceived their health as poor were more likely to change their behaviors [63]. Of note is that attrition was high in this category, with three of the studies losing roughly half of the sample between baseline and follow-up.

### Methodology and sampling

Given the heterogeneity within the field of research in terms of e.g., types of intervention, outcome measures, and psychosocial moderators, a scoping review was conducted to broadly summarize available research and identify knowledge gaps. Several important methodological issues were observed across the included studies, the first of which concerns the assessment of both psychosocial factors, and the outcome variables. (1) There are often many risk factors involved leading up to a CVD. Still, most of the studies focused on a single risk factor. In fact, only one study used a composite score assessing risk of CVD. (2) Five studies assessed moderating effects using sub-group analyses rather than interaction analysis, and thus these results should be interpreted with caution. (3) The outcomes were assessed through self-reports in most studies, which might bring some uncertainty to the reliability of the results. (4) The quality of the assessment of the psychosocial moderators is of particular importance for this review. Despite the availability of comprehensive, accessible, and validated instruments to measure psychosocial constructs, about half of the included studies did not assess psychosocial variables using validated instruments. Consequently, the use of unvalidated instruments may introduce measurement error, which in turn may lead to erroneous conclusions regarding the effect of a psychosocial factor as a moderator. Thus, moderating effects of psychosocial factors could either be inflated, or remain undetected, potentially affecting clinical recommendations and possibly the effectiveness of future interventions.

Other issues concerned the samples investigated. (1) The samples in many of the studies were homogenous in terms of demographic factors such as age, sex, and place of residence. As in all studies, this makes the sample well defined and can provide control for certain background factors, but it also contributes to the risk of type-II error and makes it more difficult to generalize results to a general population. (2) In about half of the studies, the sample consisted of participants who were either considered to be at elevated risk of CVD, or had at least one risk factor of CVD present, e.g., obesity, hypertension, sedentary lifestyle, smoking, or heavy drinking. Though it is important to study these kinds of samples in order to facilitate and investigate behavioral change amongst those most at risk, a majority of those who experience a cardiovascular event for the first time were low to moderate in CVD risk prior to the event. Therefore, interventions aimed at the general population are also of significant importance. (3) The focus of most studies has been on middle aged to older people. Only eight studies investigated samples with a mean age younger than 30 years. Of note is that all studies using alcohol consumption as an outcome were conducted on samples with a mean age < 30 years. 4) The gender distribution among the included studies was skewed, as 24 of the studies had a sample with a majority of women. 5) Most studies were conducted in Europe or North America, making it difficult to draw any general conclusions for other geographical areas. 6) In 10 studies participants were recruited through advertisements which excluded potential participants not being exposed to the advertisement. More importantly, the participants in these studies needed to have actively sought out and entered the trial. Therefore, these samples are likely to include individuals with higher motivation than a randomly selected sample. **7)** Only 14 of the studies suffered attrition rates less than 20%, and in eight studies the attrition exceeded 40%. Drop-outs from health-promoting interventions are more likely to be at higher risk and suffer worse outcomes. Therefore, the attrition rates may have consequences for the generalizability of the results.

Only five of the included studies fulfilled the criteria of being an RCT with a follow-up time of 12 months or longer and had a sample size of more than 250 participants, which in addition assessed the psychosocial moderator with a validated instrument and carried out an interaction analyses to evaluate the moderating effect. Three of these studies [49, 51, 52] investigated the moderating role of depression, whereas the other two [42, 43] evaluated the moderating effect of social support or relationship quality.

### Knowledge gaps and implications for future research

Taken together, the results from this review show a large heterogeneity in terms of study design, assessment of outcomes, potential moderators, and the samples being studied. Our findings suggest that self-efficacy may be a robust effect modifier of lifestyle interventions targeting physical activity regardless of age, intervention types or follow-up duration. However, the considerable heterogeneity among the studies limits our ability to draw definitive conclusions for most of the psychosocial factors assessed, making it difficult to translate these findings into practice. This review also highlights important gaps in the current evidence base. In particular, more research is needed regarding psychosocial factors which are not easily modifiable, e.g., depression, and regarding interventions aimed at alcohol and smoking cessation. Furthermore, we find that there is currently a lack of RCTs using (1) large samples, preferably from the general population, (2) reliable and valid instruments for assessing psychosocial variables, and (3) objectively measured outcomes, either exclusively or as a complement to self-reports. More studies fitting these criteria are needed in order to gain better understanding for the role of psychosocial factors in interventions for CVD prevention.

Compared to the number of studies on the effect of various CVD prevention and lifestyle modification programs, there is a lack of studies on the role of psychosocial factors. Despite this review finding mixed results regarding the moderating effect of psychosocial factors on lifestyle interventions, we conclude that it is important to consider these factors when planning and evaluating lifestyle interventions. However, caution is warranted when tailoring interventions based on assessed psychosocial factors. Differences in study populations and types of interventions must be carefully considered. We also observe a positive trend in an increasing number of studies investigating psychosocial moderators in relation to CVD prevention. As seen in Table 1, the number of studies that meet the criteria for inclusion in this scoping review shows more than a three-fold increase from the first decade of the century, to the second, and 13 of the included studies were published between 2020 and 2025. This may reflect an increasing awareness of the importance of considering psychosocial factors when developing and evaluating CVD prevention programs.

Within this field of research, there is a lack of studies on younger adult samples. Most studies included in this review comprised samples with participants of middle-age or older, and only nine studies included participants with a mean age below 30 years. Older age is a well-known, strong risk factor for CVD [77]. However, atherosclerosis, the main underlying mechanism for CVD, can start at a young age [78–80], as early as in a person’s 20 s [79], or even in their teens [23]. Hence, strategies for CVD-prevention should be implemented at an early age, and knowledge of psychosocial factors that may moderate adherence to lifestyle interventions differently across age groups becomes important [81].

Results from RCTs evaluating effect modification have been difficult to replicate [82]. This may be due to some studies not being powered to perform moderation analysis. Also, unequal distribution of the moderator between intervention groups may exist even after randomization, which may influence the results. Therefore, more RCTs that assess effect modification a priori are needed as well as studies that evaluate psychosocial factors as moderators of smoking and alcohol interventions.

Despite the growing popularity of online interventions, only eight of the studies investigated interventions of this format. Nevertheless, it was the most common type among studies that examined cognitive factors as potential moderators. Given their effectiveness in promoting lifestyle changes - such as increasing physical activity, improving dietary habits, and reducing smoking and alcohol consumption [83] - further research is warranted to clarify the role of psychosocial factors in the context of digital interventions specifically.

This current review focuses on psychosocial factors as moderators of effects of lifestyle interventions. However, there is another important aspect of psychosocial factors that this review does not consider, namely their role as mediators. Hence, an important topic for future research is to synthesize the evidence of psychosocial factors as mediators of lifestyle change in primary CVD prevention, in order to understand the mechanisms of health-behavior change.

### Practical implications

Identifying factors that may moderate intervention effects is a crucial step in tailoring interventions to become as effective as possible. It is therefore essential to use appropriate statistical tests to distinguish between true differences in the intervention response and chance findings. Erroneous effect modification conclusions may lead to harm when individuals are excluded from an intervention based on a wrongful notion that they may not benefit. Additionally, resources may be wasted when individuals receive an intervention that is less effective in changing their behaviors.

Previous reviews have investigated factors that influence adherence or uptake to lifestyle recommendations or intervention focused on adults that have CVD [19, 20, 22]. This review adds valuable insight into psychosocial factors that may hinder or enhance effects of lifestyle interventions in persons without diagnosed CVD. Early identification of people who may not benefit optimally from a specific lifestyle intervention is essential to adapt strategies to enhance the effectiveness of prevention efforts, and to strengthen equality in health care. In this perspective, it is important to consider whether the psychosocial moderator is modifiable or not. Moderators that are not easily modifiable would call for certain preventive strategies for certain groups. For example, the findings from the current review suggest that poor mental health, and low social support, is associated with smaller intervention effects, which calls for specific strategies for certain groups. Other moderators, such as self-efficacy, motivation and risk perception, could be addressed with the design of the intervention. When designing individually tailored interventions, researchers could consider, if practically feasible, for example a screening of relevant psychosocial factors before the start of the intervention. The purpose of the screening would be to identify psychosocial factors that may influence the success of the intervention. For instance, motivational interviewing and mastery experiences (i.e. performance accomplishments) might be used as components in the intervention to strengthen motivation and self-efficacy [84, 85]. For participants with poor mental health, tailoring may involve integrating mental health support and reducing cognitive demands of the intervention - e.g., by providing short, clear descriptions, and visual aids - since mental ill-health can impair attention, memory, and executive functioning [86].

We hope that systematic reviews with meta-analyses may be conducted in the future to investigate the strengths of the moderating effects, and in more detail chart these effects in relation to specific outcomes and to specific types of interventions. In order for that to happen, more high-quality studies are needed that a priori aim to assess moderation effects. Therefore, we would encourage those researchers designing new lifestyle interventions to consider including baseline measurements of relevant psychosocial factors using validated instruments.

### Limitations

This scoping review has several limitations that need to be addressed. Our aim was to include as many relevant psychosocial factors as possible in the search terms. However, it would have been practically impossible to include *all* possible psychosocial factors in our search. Therefore, we may have overlooked studies on the moderating effect of psychosocial factors that were not included in our search strings. In fact, two of the studies included were not identified in the searches despite fulfilling all our inclusion criteria, and were instead found through citations in articles already included.

In conducting a scoping review, our aim was to include all research on the moderating effect of psychosocial factors in interventions aimed at behaviors associated with increased CVD risk. Therefore, our search strategy was broad and resulted in a large number of unique hits. However, when scanning over 10,000 abstracts manually, studies of interest may be overlooked. In order to minimize the risk of overlooking studies for inclusion, reviewers flagged and discussed any questionable studies until consensus had been reached.

Because the psychosocial factors were person-factors, it cannot be excluded that factors correlating with the psychosocial factors can account for the moderating effects. In addition, there are likely overlaps between the categories of psychosocial moderators we created in order to systematically analyze the results. For example, some studies investigated aspects of mental health as a moderator, but mental health may also be associated with social isolation, or cognitive impairments, making it difficult to rule out effects from other psychosocial factors that were not explicitly assessed. Another point to consider is the possibility of publication bias, which occurs when results from published studies systematically differ from unpublished ones. In this scoping review, we have not assessed the likelihood of publication bias and to what extent this has affected our results. However, publication bias may lead to an exaggeration of the moderating effects of psychosocial factors.

Finally, although a majority of the studies were RCTs which adjusted for possible confounders, moderator analyses were seldom planned a priori and therefore residual confounding may remain.

### Summary and conclusionsagarmid A. Rayyan-a w

The purpose of this scoping review was to systematically chart studies that investigated psychosocial factors as moderators of lifestyle intervention for primary CVD prevention. This review highlights the heterogeneity amongst the studies in terms of e.g., the psychosocial factors investigated, types of intervention, and outcome of interest. Though our results suggest that various psychosocial factors may moderate the outcome of lifestyle interventions, this heterogeneity limited our ability to draw more detailed conclusions or identify any general trends. In order to inform intervention strategies and to improve primary prevention, more high quality RCTs are needed that a priori aim to assess moderation effects, using appropriate statistical methods and validated instruments.

## Supplementary Information

Supplementary Material 1.

Supplementary Material 2.

## Data Availability

No datasets were generated or analysed during the current study.

## Abbreviations

BCT: Behavior change techniques
BCTTv1: Behavior change techniques taxonomy
BMI: Body mass index
CVD: Cardiovascular disease
FRS: Framingham risk score
PRISMA-ScR: The preferred reporting items for systematic reviews and meta-analysis extension for scoping reviews
RCT: Randomized controlled trial
SCORE: Systematic coronary risk estimation
