# Supplementary material for: Psychosocial moderators of the effect of lifestyle interventions in primary prevention of cardiovascular disease: a scoping review
Source: BMC Public Health. 2025 Aug 30;25:2969. doi: 10.1186/s12889-025-24076-2 (PMC12398150; doi:10.1186/s12889-025-24076-2)
Supplement: Supplementary file 1 — Supplementary Material 1. [file 12889_2025_24076_MOESM1_ESM.docx]

**Detailed search string**

For an enhanced overview, the search string is divided into four sets, of which Set A relates to cardiovascular outcomes, Set B to lifestyle modifications, Set C to psychosocial factors, and Set D to research design. Each set represents an “and” criterion and several “or” criteria.

**(Set A**)**:** cvd OR "cardiovascular disease" OR "cardiovascular risk" OR "atherosclerosis" OR "myocardial infarction" OR FRS OR "Framingham risk" OR score OR score2 OR smoking OR "alcohol consumption" OR "alcohol intake" OR diet* OR "calorie intake" OR "caloric intake" OR fruit* OR vegetable* OR exercise OR "physical activity" OR sedentary OR sitting OR walking.

**AND (Set B):** "lifestyle modification" OR "lifestyle change" OR "lifestyle intervention" OR "healthy lifestyle" OR "lifestyle recommendations" OR "health behaviour change" OR “health behavior change” OR "behaviour modification" OR "behavior modification" OR “behaviour change” OR “behavior change”

**AND (Set C):** psych* OR personality OR optimism OR pessimism OR "positive affect" OR "negative affect" OR mood OR feelings OR anger OR hostility OR gratitude OR happiness OR resilience OR hope OR vitality OR resilience OR emotion* OR esteem OR confidence OR coping OR motivation* OR attachment OR helplessness OR hopelessness OR "self-efficacy" OR "self efficacy" OR “self regulation” OR “self-regulation” OR "readiness for change" OR "perceived risk" OR "risk perception" OR "post-traumatic stress disorder" OR "post traumatic stress disorder" OR ptsd OR trauma OR coping OR mastery OR "response efficacy" OR "efficacy belief" OR "efficacy beliefs" OR "expectation of outcome" OR "health-related quality of life" OR "health related quality of life" OR hrqol OR wellness OR "well-being" OR "well being" OR "health beliefs" OR "health perception" OR "health literacy" OR "perceived stress" OR "life stress" OR "life-satisfaction" OR "life satisfaction" OR distress OR depression OR worry OR anxiety OR exhaustion OR burnout OR fatigue OR "mental health" OR "body-dissatisfaction" OR "body dissatisfaction" OR "bodily dissatisfaction" OR "executive function" OR "executive functioning" OR memory OR cognition OR cognitive OR intelligence OR iq OR interpersonal OR "social support" OR "social network" OR "social networks" OR loneliness OR norms OR attitude*.

**AND (Set D):** longitudinal OR “randomized controlled trial” OR “randomized clinical trial” OR “randomized clinical trial” OR “randomised controlled trial” OR “randomised clinical trial” OR “randomised clinical trial” OR “retrospective” OR “quasi experimental”
