# Supplementary material for: Psychosocial moderators of the effect of lifestyle interventions in primary prevention of cardiovascular disease: a scoping review
Source: BMC Public Health. 2025 Aug 30;25:2969. doi: 10.1186/s12889-025-24076-2 (PMC12398150; doi:10.1186/s12889-025-24076-2)
Supplement: Supplementary file 2 — Supplementary Material 2. [file 12889_2025_24076_MOESM2_ESM.docx]

**Syntaxes for searches of each database**

**Database:** SCOPUS

**Search:** Title/Abstract

**Filters applied:** Years: 1983-2023; Document type: Article; Language: English**;** Source type: Journal

**Syntax:**

(TITLE-ABS-KEY(cvd OR "cardiovascular disease" OR "cardiovascular risk" OR "atherosclerosis" OR "myocardial infarction" OR frs OR "Framingham risk" OR score OR score2 OR smoking OR "alcohol consumption" OR "alcohol intake" OR diet* OR "calorie intake" OR "caloric intake" OR fruit* OR vegetable* OR exercise OR "physical activity" OR sedentary OR sitting OR walking) AND TITLE-ABS-KEY("lifestyle modification" OR "lifestyle change" OR "lifestyle intervention" OR "healthy lifestyle" OR "lifestyle recommendations" OR "health behaviour change" OR "health behavior change" OR "behaviour modification" OR "behavior modification" OR "behaviour change" OR "behavior change") AND TITLE-ABS-KEY(psych* OR personality OR optimism OR pessimism OR "positive affect" OR "negative affect" OR mood OR feelings OR anger OR hostility OR gratitude OR happiness OR resilience OR hope OR vitality OR resilience OR emotion* OR esteem OR confidence OR coping OR motivation* OR attachment OR helplessness OR hopelessness OR "self-efficacy" OR "self efficacy" OR "self regulation" OR "self-regulation" OR "readiness for change" OR "perceived risk" OR "risk perception" OR "post-traumatic stress disorder" OR "post traumatic stress disorder" OR ptsd OR trauma OR coping OR mastery OR "response efficacy" OR "efficacy belief" OR "efficacy beliefs" OR "expectation of outcome" OR "health-related quality of life" OR "health related quality of life" OR hrqol OR wellness OR "well-being" OR "well being" OR "health beliefs" OR "health perception" OR "health literacy" OR "perceived stress" OR "life stress" OR "life-satisfaction" OR "life satisfaction" OR distress OR depression OR worry OR anxiety OR exhaustion OR burnout OR fatigue OR "mental health" OR "body-dissatisfaction" OR "body dissatisfaction" OR "bodily dissatisfaction" OR "executive function" OR "executive functioning" OR memory OR cognition OR cognitive OR intelligence OR iq OR interpersonal OR "social support" OR "social network" OR "social networks" OR loneliness OR norms OR attitude*) AND TITLE-ABS-KEY(longitudinal OR "randomized controlled trial" OR "randomized clinical trial" OR "randomized clinical trial" OR "randomised controlled trial" OR "randomised clinical trial" OR "randomised clinical trial" OR "retrospective" OR "quasi experimental")) AND PUBYEAR > 1982 AND PUBYEAR < 2025 AND ( LIMIT-TO ( DOCTYPE,"ar" ) ) AND ( LIMIT-TO ( LANGUAGE,"English" ) ) AND ( LIMIT-TO ( SRCTYPE,"j" ) )

**Database:** Web of Science

**Search:** Topic (title, abstract, author keywords, and Keywords Plus)

**Filters applied:** Years: N/A (no hits prior to 1992); Document types: Article and early access; Language: English

**Syntax:**

cvd OR "cardiovascular disease" OR "cardiovascular risk" OR "atherosclerosis" OR "myocardial infarction" OR FRS OR "Framingham risk" OR score OR score2 OR smoking OR "alcohol consumption" OR "alcohol intake" OR diet* OR "calorie intake" OR "caloric intake" OR fruit* OR vegetable* OR exercise OR "physical activity" OR sedentary OR sitting OR walking (Topic) and "lifestyle modification" OR "lifestyle change" OR "lifestyle intervention" OR "healthy lifestyle" OR "lifestyle recommendations" OR "health behaviour change" OR “health behavior change” OR "behaviour modification" OR "behavior modification" OR “behaviour change” OR “behavior change” (Topic) and psych* OR personality OR optimism OR pessimism OR "positive affect" OR "negative affect" OR mood OR feelings OR anger OR hostility OR gratitude OR happiness OR resilience OR hope OR vitality OR resilience OR emotion* OR esteem OR confidence OR coping OR motivation* OR attachment OR helplessness OR hopelessness OR "self-efficacy" OR "self efficacy" OR “self regulation” OR “self-regulation” OR "readiness for change" OR "perceived risk" OR "risk perception" OR "post-traumatic stress disorder" OR "post traumatic stress disorder" OR ptsd OR trauma OR coping OR mastery OR "response efficacy" OR "efficacy belief" OR "efficacy beliefs" OR "expectation of outcome" OR "health-related quality of life" OR "health related quality of life" OR hrqol OR wellness OR "well-being" OR "well being" OR "health beliefs" OR "health perception" OR "health literacy" OR "perceived stress" OR "life stress" OR "life-satisfaction" OR "life satisfaction" OR distress OR depression OR worry OR anxiety OR exhaustion OR burnout OR fatigue OR "mental health" OR "body-dissatisfaction" OR "body dissatisfaction" OR "bodily dissatisfaction" OR "executive function" OR "executive functioning" OR memory OR cognition OR cognitive OR intelligence OR iq OR interpersonal OR "social support" OR "social network" OR "social networks" OR loneliness OR norms OR attitude* (Topic) and longitudinal OR “randomized controlled trial” OR “randomized clinical trial” OR “randomized clinical trial” OR “randomised controlled trial” OR “randomised clinical trial” OR “randomised clinical trial” OR “retrospective” OR “quasi experimental” (Topic) and Early Access or Article (Document Types) and English (Languages)

**Database:** PubMed

**Search:** Article title, Abstract, Keywords

**Filters applied:** Years: N/A (oldest hit 1983); Text Availability: Full text**;** Language: English

**Syntax:**

(((cvd[Title/Abstract] OR "cardiovascular disease"[Title/Abstract] OR "cardiovascular risk"[Title/Abstract] OR "atherosclerosis"[Title/Abstract] OR "myocardial infarction"[Title/Abstract] OR FRS[Title/Abstract] OR "Framingham risk"[Title/Abstract] OR score[Title/Abstract] OR score2[Title/Abstract] OR smoking[Title/Abstract] OR "alcohol consumption"[Title/Abstract] OR "alcohol intake"[Title/Abstract] OR diet*[Title/Abstract] OR "calorie intake"[Title/Abstract] OR "caloric intake"[Title/Abstract] OR fruit*[Title/Abstract] OR vegetable*[Title/Abstract] OR exercise[Title/Abstract] OR "physical activity"[Title/Abstract] OR sedentary[Title/Abstract] OR sitting[Title/Abstract] OR walking[Title/Abstract]) AND ("lifestyle modification"[Title/Abstract] OR "lifestyle change"[Title/Abstract] OR "lifestyle intervention"[Title/Abstract] OR "healthy lifestyle"[Title/Abstract] OR "lifestyle recommendations"[Title/Abstract] OR "health behaviour change"[Title/Abstract] OR "health behavior change"[Title/Abstract] OR "behaviour modification"[Title/Abstract] OR "behavior modification"[Title/Abstract] OR "behaviour change"[Title/Abstract] OR "behavior change"[Title/Abstract])) AND (psych*[Title/Abstract] OR personality[Title/Abstract] OR optimism[Title/Abstract] OR pessimism[Title/Abstract] OR "positive affect"[Title/Abstract] OR "negative affect"[Title/Abstract] OR mood[Title/Abstract] OR feelings[Title/Abstract] OR anger[Title/Abstract] OR hostility[Title/Abstract] OR gratitude[Title/Abstract] OR happiness[Title/Abstract] OR resilience[Title/Abstract] OR hope[Title/Abstract] OR vitality[Title/Abstract] OR resilience[Title/Abstract] OR emotion*[Title/Abstract] OR esteem[Title/Abstract] OR confidence[Title/Abstract] OR coping[Title/Abstract] OR motivation*[Title/Abstract] OR attachment[Title/Abstract] OR helplessness[Title/Abstract] OR hopelessness[Title/Abstract] OR "self-efficacy"[Title/Abstract] OR "self efficacy"[Title/Abstract] OR "self regulation"[Title/Abstract] OR "self-regulation"[Title/Abstract] OR "readiness for change"[Title/Abstract] OR "perceived risk"[Title/Abstract] OR "risk perception"[Title/Abstract] OR "post-traumatic stress disorder"[Title/Abstract] OR "post traumatic stress disorder"[Title/Abstract] OR ptsd[Title/Abstract] OR trauma[Title/Abstract] OR coping[Title/Abstract] OR mastery[Title/Abstract] OR "response efficacy"[Title/Abstract] OR "efficacy belief"[Title/Abstract] OR "efficacy beliefs"[Title/Abstract] OR "expectation of outcome"[Title/Abstract] OR "health-related quality of life"[Title/Abstract] OR "health related quality of life"[Title/Abstract] OR hrqol[Title/Abstract] OR wellness[Title/Abstract] OR "well-being"[Title/Abstract] OR "well being"[Title/Abstract] OR "health beliefs"[Title/Abstract] OR "health perception"[Title/Abstract] OR "health literacy"[Title/Abstract] OR "perceived stress"[Title/Abstract] OR "life stress"[Title/Abstract] OR "life-satisfaction"[Title/Abstract] OR "life satisfaction"[Title/Abstract] OR distress[Title/Abstract] OR depression[Title/Abstract] OR worry[Title/Abstract] OR anxiety[Title/Abstract] OR exhaustion[Title/Abstract] OR burnout[Title/Abstract] OR fatigue[Title/Abstract] OR "mental health"[Title/Abstract] OR "body-dissatisfaction"[Title/Abstract] OR "body dissatisfaction"[Title/Abstract] OR "bodily dissatisfaction"[Title/Abstract] OR "executive function"[Title/Abstract] OR "executive functioning"[Title/Abstract] OR memory[Title/Abstract] OR cognition[Title/Abstract] OR cognitive[Title/Abstract] OR intelligence[Title/Abstract] OR iq[Title/Abstract] OR interpersonal[Title/Abstract] OR "social support"[Title/Abstract] OR "social network"[Title/Abstract] OR "social networks"[Title/Abstract] OR loneliness[Title/Abstract] OR norms[Title/Abstract] OR attitude*[Title/Abstract])) AND (longitudinal[Title/Abstract] OR "randomized controlled trial"[Title/Abstract] OR "randomized clinical trial"[Title/Abstract] OR "randomized clinical trial"[Title/Abstract] OR "randomised controlled trial"[Title/Abstract] OR "randomised clinical trial"[Title/Abstract] OR "randomised clinical trial"[Title/Abstract] OR "retrospective"[Title/Abstract] OR "quasi experimental"[Title/Abstract])

**Database:** APA PsycInfo

**Filters applied:** Years: N/A, no hits prior to 1991; Type: Academic journals; Language: English

**Syntax:**

AB (cvd OR "cardiovascular disease" OR "cardiovascular risk" OR "atherosclerosis" OR "myocardial infarction" OR FRS OR "Framingham risk" OR score OR score2 OR smoking OR "alcohol consumption" OR "alcohol intake" OR diet* OR "calorie intake" OR "caloric intake" OR fruit* OR vegetable* OR exercise OR "physical activity" OR sedentary OR sitting OR walking ) OR TI ( cvd OR "cardiovascular disease" OR "cardiovascular risk" OR "atherosclerosis" OR "myocardial infarction" OR FRS OR "Framingham risk" OR score OR score2 OR smoking OR "alcohol consumption" OR "alcohol intake" OR diet* OR "calorie intake" OR "caloric intake" OR fruit* OR vegetable* OR exercise OR "physical activity" OR sedentary OR sitting OR walking ) OR KW ( cvd OR "cardiovascular disease" OR "cardiovascular risk" OR "atherosclerosis" OR "myocardial infarction" OR FRS OR "Framingham risk" OR score OR score2 OR smoking OR "alcohol consumption" OR "alcohol intake" OR diet* OR "calorie intake" OR "caloric intake" OR fruit* OR vegetable* OR exercise OR "physical activity" OR sedentary OR sitting OR walking ) OR SU ( cvd OR "cardiovascular disease" OR "cardiovascular risk" OR "atherosclerosis" OR "myocardial infarction" OR FRS OR "Framingham risk" OR score OR score2 OR smoking OR "alcohol consumption" OR "alcohol intake" OR diet* OR "calorie intake" OR "caloric intake" OR fruit* OR vegetable* OR exercise OR "physical activity" OR sedentary OR sitting OR walking ) AND AB ( "lifestyle modification" OR "lifestyle change" OR "lifestyle intervention" OR "healthy lifestyle" OR "lifestyle recommendations" OR "health behaviour change" OR “health behavior change” OR "behaviour modification" OR "behavior modification" OR “behaviour change” OR “behavior change” ) OR TI ( "lifestyle modification" OR "lifestyle change" OR "lifestyle intervention" OR "healthy lifestyle" OR "lifestyle recommendations" OR "health behaviour change" OR “health behavior change” OR "behaviour modification" OR "behavior modification" OR “behaviour change” OR “behavior change” ) OR KW ( "lifestyle modification" OR "lifestyle change" OR "lifestyle intervention" OR "healthy lifestyle" OR "lifestyle recommendations" OR "health behaviour change" OR “health behavior change” OR "behaviour modification" OR "behavior modification" OR “behaviour change” OR “behavior change” ) OR SU ( "lifestyle modification" OR "lifestyle change" OR "lifestyle intervention" OR "healthy lifestyle" OR "lifestyle recommendations" OR "health behaviour change" OR “health behavior change” OR "behaviour modification" OR "behavior modification" OR “behaviour change” OR “behavior change” ) AND AB ( psych* OR personality OR optimism OR pessimism OR "positive affect" OR "negative affect" OR mood OR feelings OR anger OR hostility OR gratitude OR happiness OR resilience OR hope OR vitality OR resilience OR emotion* OR esteem OR confidence OR coping OR motivation* OR attachment OR helplessness OR hopelessness OR "self-efficacy" OR "self efficacy" OR “self regulation” OR “self-regulation” OR "readiness for change" OR "perceived risk" OR "risk perception" OR "post-traumatic stress disorder" OR "post traumatic stress disorder" OR ptsd OR trauma OR coping OR mastery OR "response efficacy" OR "efficacy belief" OR "efficacy beliefs" OR "expectation of outcome" OR "health-related quality of life" OR "health related quality of life" OR hrqol OR wellness OR "well-being" OR "well being" OR "health beliefs" OR "health perception" OR "health literacy" OR "perceived stress" OR "life stress" OR "life-satisfaction" OR "life satisfaction" OR distress OR depression OR worry OR anxiety OR exhaustion OR burnout OR fatigue OR "mental health" OR "body-dissatisfaction" OR "body dissatisfaction" OR "bodily dissatisfaction" OR "executive function" OR "executive functioning" OR memory OR cognition OR cognitive OR intelligence OR iq OR interpersonal OR "social support" OR "social network" OR "social networks" OR loneliness OR norms OR attitude* ) OR TI ( psych* OR personality OR optimism OR pessimism OR "positive affect" OR "negative affect" OR mood OR feelings OR anger OR hostility OR gratitude OR happiness OR resilience OR hope OR vitality OR resilience OR emotion* OR esteem OR confidence OR coping OR motivation* OR attachment OR helplessness OR hopelessness OR "self-efficacy" OR "self efficacy" OR “self regulation” OR “self-regulation” OR "readiness for change" OR "perceived risk" OR "risk perception" OR "post-traumatic stress disorder" OR "post traumatic stress disorder" OR ptsd OR trauma OR coping OR mastery OR "response efficacy" OR "efficacy belief" OR "efficacy beliefs" OR "expectation of outcome" OR "health-related quality of life" OR "health related quality of life" OR hrqol OR wellness OR "well-being" OR "well being" OR "health beliefs" OR "health perception" OR "health literacy" OR "perceived stress" OR "life stress" OR "life-satisfaction" OR "life satisfaction" OR distress OR depression OR worry OR anxiety OR exhaustion OR burnout OR fatigue OR "mental health" OR "body-dissatisfaction" OR "body dissatisfaction" OR "bodily dissatisfaction" OR "executive function" OR "executive functioning" OR memory OR cognition OR cognitive OR intelligence OR iq OR interpersonal OR "social support" OR "social network" OR "social networks" OR loneliness OR norms OR attitude* ) OR KW ( psych* OR personality OR optimism OR pessimism OR "positive affect" OR "negative affect" OR mood OR feelings OR anger OR hostility OR gratitude OR happiness OR resilience OR hope OR vitality OR resilience OR emotion* OR esteem OR confidence OR coping OR motivation* OR attachment OR helplessness OR hopelessness OR "self-efficacy" OR "self efficacy" OR “self regulation” OR “self-regulation” OR "readiness for change" OR "perceived risk" OR "risk perception" OR "post-traumatic stress disorder" OR "post traumatic stress disorder" OR ptsd OR trauma OR coping OR mastery OR "response efficacy" OR "efficacy belief" OR "efficacy beliefs" OR "expectation of outcome" OR "health-related quality of life" OR "health related quality of life" OR hrqol OR wellness OR "well-being" OR "well being" OR "health beliefs" OR "health perception" OR "health literacy" OR "perceived stress" OR "life stress" OR "life-satisfaction" OR "life satisfaction" OR distress OR depression OR worry OR anxiety OR exhaustion OR burnout OR fatigue OR "mental health" OR "body-dissatisfaction" OR "body dissatisfaction" OR "bodily dissatisfaction" OR "executive function" OR "executive functioning" OR memory OR cognition OR cognitive OR intelligence OR iq OR interpersonal OR "social support" OR "social network" OR "social networks" OR loneliness OR norms OR attitude* ) OR SU ( psych* OR personality OR optimism OR pessimism OR "positive affect" OR "negative affect" OR mood OR feelings OR anger OR hostility OR gratitude OR happiness OR resilience OR hope OR vitality OR resilience OR emotion* OR esteem OR confidence OR coping OR motivation* OR attachment OR helplessness OR hopelessness OR "self-efficacy" OR "self efficacy" OR “self regulation” OR “self-regulation” OR "readiness for change" OR "perceived risk" OR "risk perception" OR "post-traumatic stress disorder" OR "post traumatic stress disorder" OR ptsd OR trauma OR coping OR mastery OR "response efficacy" OR "efficacy belief" OR "efficacy beliefs" OR "expectation of outcome" OR "health-related quality of life" OR "health related quality of life" OR hrqol OR wellness OR "well-being" OR "well being" OR "health beliefs" OR "health perception" OR "health literacy" OR "perceived stress" OR "life stress" OR "life-satisfaction" OR "life satisfaction" OR distress OR depression OR worry OR anxiety OR exhaustion OR burnout OR fatigue OR "mental health" OR "body-dissatisfaction" OR "body dissatisfaction" OR "bodily dissatisfaction" OR "executive function" OR "executive functioning" OR memory OR cognition OR cognitive OR intelligence OR iq OR interpersonal OR "social support" OR "social network" OR "social networks" OR loneliness OR norms OR attitude* ) AND AB ( longitudinal OR “randomized controlled trial” OR “randomized clinical trial” OR “randomized clinical trial” OR “randomised controlled trial” OR “randomised clinical trial” OR “randomised clinical trial” OR “retrospective” OR “quasi experimental” ) OR TI ( longitudinal OR “randomized controlled trial” OR “randomized clinical trial” OR “randomized clinical trial” OR “randomised controlled trial” OR “randomised clinical trial” OR “randomised clinical trial” OR “retrospective” OR “quasi experimental” ) OR KW ( longitudinal OR “randomized controlled trial” OR “randomized clinical trial” OR “randomized clinical trial” OR “randomised controlled trial” OR “randomised clinical trial” OR “randomised clinical trial” OR “retrospective” OR “quasi experimental” ) OR SU ( longitudinal OR “randomized controlled trial” OR “randomized clinical trial” OR “randomized clinical trial” OR “randomised controlled trial” OR “randomised clinical trial” OR “randomised clinical trial” OR “retrospective” OR “quasi experimental” )
